# High-rate pacing suppresses Torsade de Pointes arrhythmias and reduces spatial dispersion of repolarization in the chronic AV-block dog model

**DOI:** 10.3389/fphys.2023.1330230

**Published:** 2023-12-20

**Authors:** Vera Loen, Valerie Y. H. Van Weperen, Henriëtte D. M. Beekman, Joanne J. A. Van Bavel, Veronique M. F. Meijborg, Jeanne G. Van der Waal, Ruben Coronel, Marcel A. G. van der Heyden, Marc A. Vos

**Affiliations:** ^1^ Department of Medical Physiology, University Medical Center Utrecht, Utrecht, Netherlands; ^2^ Department of Clinical and Experimental Cardiology, Academic Medical Center, Amsterdam, Netherlands

**Keywords:** CAVB dog model, sudden cardiac death, high-rate pacing, spatial dispersion of repolarization, arrhythmogenesis, Torsade de Pointes

## Abstract

**Background:** An electrical storm of Torsade de Pointes arrhythmias (TdP) can be reproducibly induced in the anesthetized chronic AV-block (CAVB) dog by infusion of the I_Kr_-blocker dofetilide. Earlier studies showed that these arrhythmias 1) arise from locations with high spatial dispersion in repolarization (SDR) and 2) can be suppressed by high-rate pacing. We examined whether suppression of TdP by high-rate pacing is established through a decrease in SDR in the CAVB dog.

**Methods:** Dofetilide (25 μg/kg in 5 min) was administered to 5 anesthetized CAVB dogs to induce TdP arrhythmias. During the experiments, animals were continuously paced from the right ventricular apex at 50 beats/minute (RVA50). Upon TdP occurrence and conversion, RVA pacing was consecutively set to 100, 80 and 60 beats/minute for 2 min, referred to as pacing blocks. To determine the additional anti-arrhythmic effects of HRP over defibrillation alone, the number of arrhythmic events and SDR at RVA100 were compared to data from three previously conducted experiments, in which dogs underwent the same experimental protocol but were paced at RVA60 upon TdP occurrence (RVA60_retro_). In all experiments, recordings included surface electrocardiogram and mapping by 56 intramural needles, each recording four electrograms, evenly inserted into the ventricular walls and septum. For each pacing block, the number of ectopic beats (EB), and TdP severity were scored. SDR was quantified as the average difference in repolarization time within four squared needles (SDR_cubic_).

**Results:** In 4 out of 5 animals, pacing at RVA100 suppressed TdP occurrence. One dog could not be converted by defibrillation after the initial TdP. Compared to RVA50, pacing at RVA100, but not RVA80 and RVA60, significantly reduced the TdP score (78 ± 33 vs*.* 0 ± 0, *p* < 0.05 and vs*.* 12.5 ± 25 and 25 ± 50, both *p* > 0.05). The reduction in TdP score was reflected by a significant decrease in SDR_cubic_ (125 ± 46 ms before TdP vs*.* 49 ± 18 ms during RVA100, *p* < 0.05), and SDR was smaller than in the RVA60_retro_ animals (101 ± 52 ms, *p* < 0.05 vs*.* RVA100).

**Conclusion:** In CAVB dogs, high-rate pacing effectively suppresses TdP, which, at least in part, results from a spatial homogenization of cardiac repolarization, as reflected by a decrease in SDR.

## Introduction

Sudden cardiac death (SCD) poses a major burden on the Western healthcare system as it is a leading cause of death and heavily impacts family lives (Deo and Albert, 2012; Kumar et al., 2021). Given that ventricular arrhythmias are the primary culprits behind SCD, understanding the underlying pathophysiology and investigating potential strategies for preventing these arrhythmias continue to be important areas of research (Al-Khatib et al., 2018).

The canine Chronic Atrio-Ventricular Block (CAVB) model has proved to be extremely valuable in studies regarding SCD, as an electrical storm of Torsade de Pointes arrhythmias (TdP) can be reliably (75%–80%) and reproducibly evoked (Loen et al., 2022). The TdP-sensitivity results from the creation of a complete atrio-ventricular block by ablation of the proximal His bundle. The subsequent drop in heartrate, combined with an altered ventricular activation pattern, instigates a complex ventricular remodeling processes that restores cardiac output, but impairs the redundancy in repolarizing currents known as the ‘repolarization reserve’ (Thomsen et al., 2007). Together, these adaptations adversely lead to a high susceptibility for TdP when the repolarization machinery is additionally challenged, e.g., by anesthesia and/or pharmacological block of repolarizing currents (Vos et al., 1998; Dunnink et al., 2012).

Previous studies have identified several important factors that promote arrhythmogenesis in the CAVB dog model by encouraging triggered activity and/or modulating the arrhythmogenic substrate. Triggered activity, resulting from early or delayed after depolarizations, has been shown to initiate TdP (Vandersickel et al., 2017; Smoczynska et al., 2021). These after-depolarizations arise as a result of the diminished repolarization reserve, rendering cardiomyocytes unable to compensate for additional repolarization impediments (Varro and Baczko, 2011). Temporal variability in repolarization, quantified as short-term variability (STV) of repolarization, reflects the state of the repolarization reserve (Thomsen et al., 2004; Varro and Baczko, 2011). STV has been demonstrated to increase leading up to an episode of TdP in the CAVB dog (Varkevisser et al., 2012; Wijers et al., 2017), in pigs with cardiac ischemia (Amoni et al., 2022) as well as before spontaneously occurring ventricular arrhythmias in patients (Smoczynska et al., 2020b). Secondly, TdP arise from a location with a high spatial dispersion repolarization (SDR), and insusceptibility for TdP is associated with the absence of such regions of high SDR (Dunnink et al., 2017). Whereas triggered activity initiates arrhythmic episodes, advancement to more critical arrhythmias hinges on the presence of adequate SDR (Dunnink et al., 2017; Smoczynska et al., 2021). Finally, high-rate pacing (HRP) is a well-established method to suppress and prevent arrhythmias in both the CAVB dog model (Wijers et al., 2017; Smoczynska et al., 2020a) and patients (Eldar et al., 1987; Fisher et al., 1987; Gronefeld et al., 2002). HRP exerts its anti-arrhythmic effects by increasing the delayed rectifier potassium current and, subsequently, bolstering the repolarization reserve (Damiano and Rosen, 1984; Szentandrassy et al., 2015). Correspondingly, HRP reduces STV in CAVB dogs challenged with the specific I_Kr_ blocker dofetilide (Wijers et al., 2017; Smoczynska et al., 2020a). Moreover, pacing at lower rates was shown to increase spatial heterogeneity of repolarization (van Weperen et al., 2021). However, these SDR measurements were conducted in absence of a pharmacological challenge. Therefore, the objective of the current study was to examine whether suppression of dofetilide evoked TdP by high-rate pacing is reflected by a decrease in SDR. Thus, five CAVB dogs with inducible TdP were subjected to a proarrhythmic challenge with the specific I_Kr_ blocker dofetilide, whilst continually paced at 50 beats per minute (bpm) from the right ventricular apex (RVA50). Upon the first TdP, pacing frequency was increased to RVA100 for 2 min, followed by RVA80 and RVA60. To isolate the anti-arrhythmic effects of HRP from defibrillation, arrhythmic outcome and electrophysiological parameters were compared to three animals from previously conducted experiments that were paced at RVA60 after first TdP occurrence (RVA60_retro_). Detailed *in vivo* mapping was performed to assess the effect of different pacing rates on SDR and to correlate SDR with arrhythmic outcomes. This study shows that high-rate pacing effectively suppresses TdP in association with a homogenization of repolarization.

## Materials and methods

Animal handling and care were in accordance with the Directive 2010/63/EU of the European Parliament and of the Council of 22 September 2010 on the protection of animals used for scientific purposes and the Dutch law, laid down in the Experiments on Animals Act. The Animal Experiment Committee of the University of Utrecht approved all experiments.

The prospective part of the study included a total of five adult purpose-bred mongrel dogs (Marshall, NY; three males, two females; weight 25 ± 4 kg). For the retrospective data, three control dogs were selected (two females, one male, weight 24 ± 6 kg, see below). Dogs were housed in conventional kennels with wooden bedding, had free access to water and received food pellets twice a day. Each cage was supplied with playing tools, and animals were let out of the kennel at least once a day to go out and play in a group. Daily checks on health and comfort were performed and weight was measured once a week.

### Animal preparation

Dogs were fasted overnight and received premedication (0.02 mg/kg atropine, 0.5 mg/kg methadone, and 0.5 mg/kg acepromazine i.m.) 30 min before the surgical procedure. General anesthesia was induced by sodium pentobarbital (Nembutal, 25 mg/kg i.v.) and maintained by 1.5% isoflurane in O_2_ and N_2_O (1:2 ratio) via mechanical ventilation at 12 breaths/min. Additionally, dogs received analgesics (0.1 mg/kg Metacam s.c. before and 0.3 mg Temgesic i.m. after surgery) and antibiotics (1,000 mg ampicillin i.v. before and i.m. after surgery). During a previous procedure, a screw-in lead was advanced to the right ventricular apex (RVA) via the jugular vein and connected to an internal pacemaker (Medtronic, Maastricht, Netherlands). The proximal His-bundle was then ablated using Radiofrequency to induce complete AV-block (Oros et al., 2008). The experiments were performed after 92 ± 30 days of remodeling under idioventricular rhythm, allowing complete electrical remodeling.

### Experimental protocol

**FIGURE 1:**
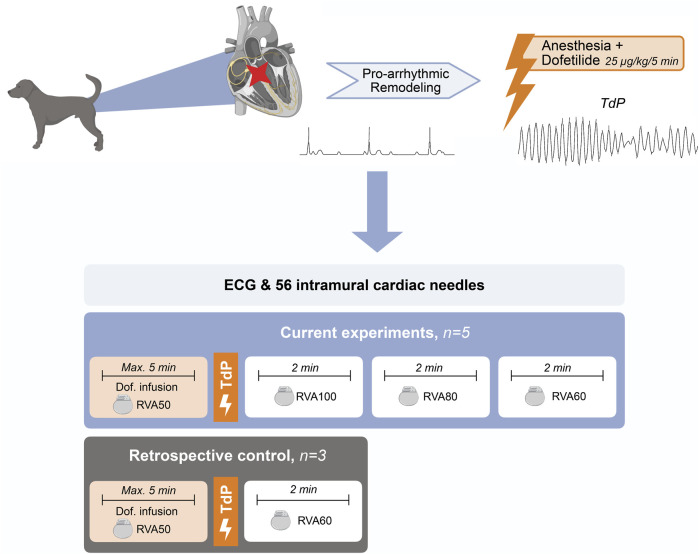
Schematic overview of experimental setup. TdP, Torsade de Pointes; Max, maximum; min, minute; Dof, dofetilide; RVA, right ventricular apical pacing.

Dogs were placed in a right lateral position. A standard six-lead ECG with four additional precordial leads (EPTracer, CardioTek, Maastricht) and an external defibrillator were applied for monitoring and safety. The internal pacemaker was set to RVA pacing at 50 bpm. Thoracotomy was performed and a total of 56 intracardiac needles, each containing four unipolar recording electrodes, were evenly distributed over the septum, left ventricular (LV) and right ventricular (RV) walls, distributed over six horizontal levels (Dunnink et al., 2017). A reference electrode was placed in the dermis near the thorax incision. The unipolar electrograms derived from the intracardiac needles were recorded with the ActiveTwo system (Biosemi, Amsterdam, sampling frequency 2048 Hz, bandwidth [-3dB] DC-400 Hz). After a stabilization period of 60 min to mitigate injury currents on the needle electrograms, dofetilide was administrated (0.025 mg/kg iv) over 5 min or until the first TdP occurred. TdP was defined as a polymorphic ventricular tachycardia characterized by at least five consecutive ectopic beats (EBs) with a twisting shape of the QRS complex around the isoelectric line. TdP lasting >10 s were terminated by electrical cardioversion, applied via thoracic paddles connected to the external defibrillator. After the occurrence of a TdP, the pacing frequency was increased to RVA100 for 2 min, followed by RVA80 for 2 min and finally RVA60 for 2 min (Figure 1).

### Data analysis

Electrophysiological parameters were measured just before the first EB and TdP following dofetilide infusion (both RVA50), and 60 s after initiation of RVA100, RVA80 and RVA60 pacing. Using EPTracer software, RR and QT intervals were measured from lead II of the surface ECG and averaged over five consecutive beats. QT interval was corrected for heart rate (QTc) using the Van de Water formula, since this correction was optimized for use in anesthetized dogs specifically and proved more adequate than standard correction formulas (Van de Water et al., 1989). Unipolar electrograms were analyzed using custom-made Matlab software (Mathworks, Natick, MA) (Potse et al., 2002). Needle recordings showing persistent major injury current (>80% of the T-wave amplitude) despite stabilization, noise, flat T-waves, or incorrect placement of the needle protruding into the ventricle (cavity potentials) were disregarded. Activation time (AT) was defined as the time of minimum dV/dt of the QRS complex and repolarization time (RT) as the time of maximum dV/dt of the T wave irrespective of the T-wave morphology. SDR was calculated from the maximum RT differences in transmural, horizontal, vertical, and cubic orientation and averaged for each orientation to quantify the four SDR directions per dog (Dunnink et al., 2017). SDR was visualized in polar maps after correction of individual needle RTs by the Van de Water formula, aiming to attenuate the confounding effect of different heart rates on repolarization time. Subsequently, polar maps were generated using Adobe Illustrator CS6 (Adobe Systems, San José, CA). Restitution curves for repolarization were generated using the averaged LV and RV RTs of the unipolar electrograms and corresponding RR-intervals.

For arrhythmia quantification, the total number of ectopic beats and TdP episodes were counted in each pacing block. During dofetilide infusion (RVA50), only the last 2 min before a TdP occurred were considered. For quantification of the severity of the TdP episodes, a TdP score was calculated as follows. For a self-terminating TdP, the number of beats in the episode represent the TdP score, while non self-terminating TdP were awarded for 50, 75 or 100 points when requiring 1, 2 or ≥3 defibrillations respectively. When >1 TdP occurred in one pacing block, the most severe episode was scored.

### Retrospective control

To assess the additional anti-arrhythmic effect of RVA100 pacing compared to defibrillation alone, SDR at RVA100 was compared to previously performed *in vivo* mapping experiments in which animals were paced at RVA60 after defibrillation. The database containing *in vivo* mapping data was screened for previous experiments with a similar remodeling duration (119 ± 30 days), RVA60 for 2 minutes after defibrillation, absence of arrhythmia-modulating factors other than dofetilide and the same anesthesiology regimen. This search yielded three eligible dogs. The same electrophysiological parameters from the ECG and needle electrodes were measured as described above and were calculated at 1 minute after defibrillation to compare to the current work’s results.

### Statistical analysis

Data are expressed as mean ± standard deviation (SD) or median (interquartile range (IQR)), unless indicated otherwise. Serial comparison of electrophysiological parameters was performed with a mixed analysis of variance (ANOVA), with *post hoc* Tukey correction for multiple comparisons. TdP score and number of ectopic beats were analyzed using the Kruskal–Wallis test, with *post hoc* Dunn correction for multiple comparisons. Differences were considered significant when *p* < 0.05. Statistical analysis was performed using GraphPad Prism 10.0 (GraphPad Software Inc., La Jolla, CA, United States).

## Results

**FIGURE 2:**
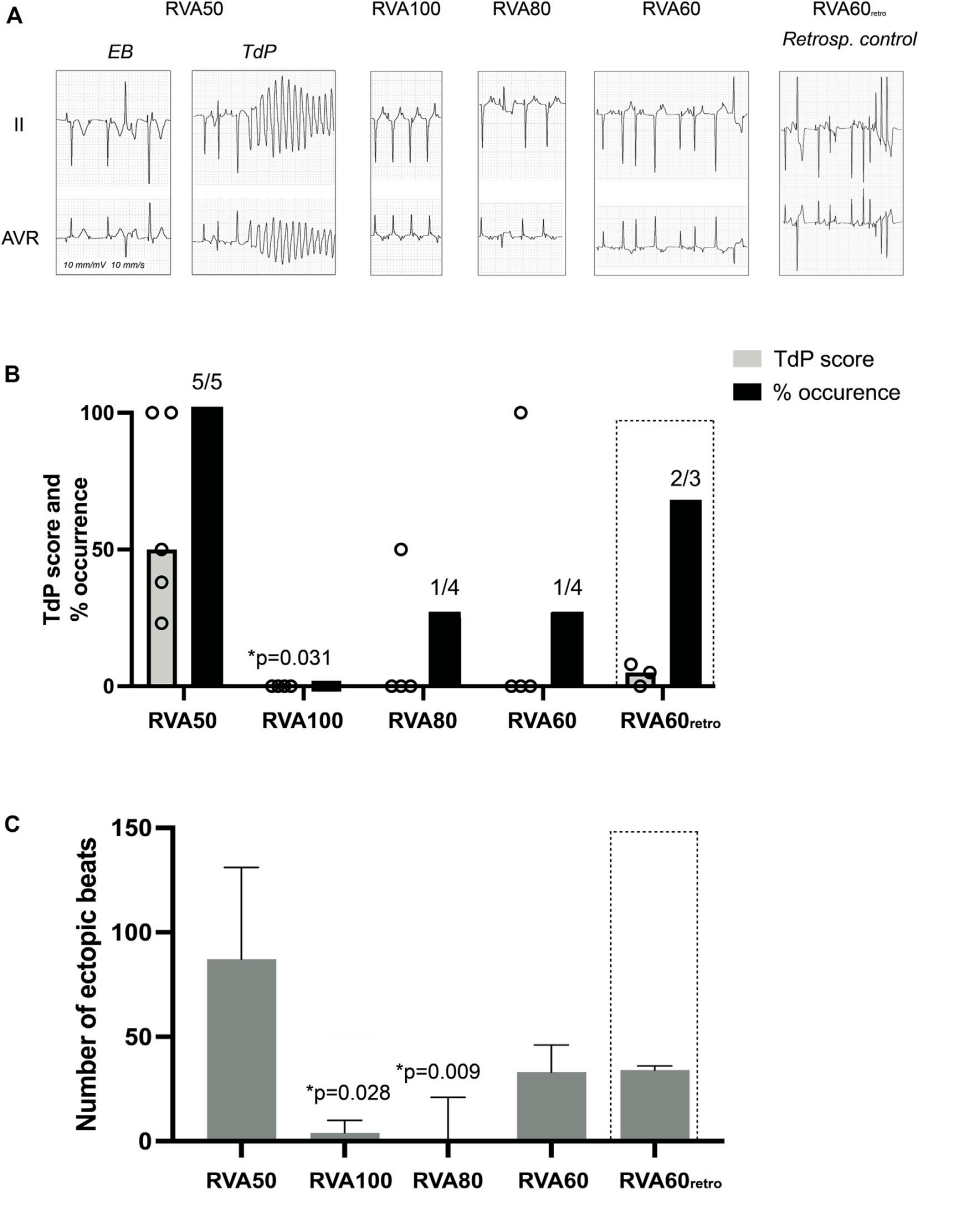
Effect of different RVA pacing rates on arrhythmic outcomes after dofetilide infusion in chronic atrioventricular block dogs. **(A)** Representative ECG traces (lead II and aVR) after 5 μg/kg per 5 min dofetilide infusion during different RVA pacing rates. **(B)** Incidence of TdP occurrence and TdP score, defined as the number of beats or 50/75/100 points when requiring 1, 2 or ≥3 defibrillations respectively of the most severe episode during different RVA pacing rates. **(C)** Cumulative number of ectopic beats during different RVA pacing rates. Values represented as mean ± SD unless indicated otherwise. EB, ectopic beat; TdP, Torsade de Pointes arrhythmia; RVA, right ventricular apical pacing. *p < 0.05 vs. RVA50.

One dog could not be resuscitated after the initial TdP, leaving 4 dogs for measurements during RVA100, RVA80 and RVA60 pacing. On average, the first EB occurred 181 ± 60 s after dofetilide infusion, followed by TdP 242 ± 75 s after infusion. Four out of five of the TdPs during dofetilide infusion required defibrillation, producing a median TdP score of 50 (77) (Figures 2A, B; Table 1). RVA100 pacing prevented TdP completely (TdP score 0 (0), *p* = 0.031). Moreover, the total number of ectopic beats during the 2-min pacing blocks decreases significantly from RVA50 (87 (104)) to higher pacing rates (Figure 2C; Table 1).

**TABLE 1:** Serial comparison of electrophysiological effect of different pacing rates following dofetilide administration.

	RVA50		RVA100	RVA80	RVA60	RVA60_retro_
Parameters	EB	TdP				Retrospective control
n	5	5	4	4	4	3
RR (ms)	1,200 ± 0	1,200 ± 0	600 ± 0	750 ± 0	1,000 ± 0	1,000 ± 0
QT (ms)	573 ± 51	626 ± 78	453 ± 15*^+^	508 ± 19^+^	598 ± 29^§^^	513 ± 64^+^
QTc (ms)	555 ± 51	609 ± 79	488 ± 15^+^	530 ± 18^§^	598 ± 29^§^	513 ± 64
RV-AT (ms) needles	40 ± 14	41 ± 18	39 ± 12	39 ± 12	38 ± 12	42 ± 16
LV-AT (ms) needles	63 ± 17	62 ± 18	60 ± 17	60 ± 16	60 ± 16	59 ± 21
RV-RT (ms) needles	363 ± 54	403 ± 73*	330 ± 25*^+^	376 ± 32^+§^	392 ± 46*^§^	360 ± 27^+§†^
LV-RT (ms) needles	453 ± 80	480 ± 74*	363 ± 25*^+^	413 ± 28*^+§^	464 ± 54*^+§^^	395 ± 50*^+§^†^
dRT	90 ± 66	78 ± 81	33 ± 27*^+^	37 ± 35*^+^	73 ± 54^*§*^ *^*	35 ± 40*^+†^
Transmural dispersion (ms)	48 ± 33	62 ± 46*	25 ± 18*^+^	30 ± 21*^+^	48 ± 31^+§^^	49 ± 32^+§^^
Vertical dispersion (ms)	69 ± 37	74 ± 50	32 ± 18*^+^	47 ± 23*^+^	66 ± 32^§^^	59 ± 51^§^
Horizontal dispersion (ms)	78 ± 37	98 ± 48*	38 ± 18*^+^	46 ± 22*^+^	75 ± 33^+§^^	75 ± 43^+§^^
Cubic dispersion (ms)	103 ± 32	125 ± 46*	49 ± 18*^+^	62 ± 24*^+^	107 ± 29^+§^^	101 ± 52^+§^^
TdP occurrence %	NA	100	0	25	25	66
Median n TdP	NA	1 (1)	0 (0)	0 (1)	0 (3)	1 (2)
TdP score	50 (77) ^§^		0 (0)	0 (50)	0 (100)	5 (8)
Median n ectopy	87 (104)^§^^		4 (10)	0 (21)	33 (46)	34 (5)

Values are represented as mean ± SD, or median (IQR), unless indicated otherwise. *p < 0.05 vs. EB, ^+^ *p* < 0.05 vs. TdP, ^§^ *p* < 0.05 vs. RVA100, ^*p* < 0.05 vs. RVA80, ^†^ *p* < 0.05 vs. RVA60. RV-AT, right ventricular activation time; LV-AT, left ventricular activation time; RV-RT, right ventricular repolarization time; LV-RT, left ventricular repolarization time; dRT, delta RT; TdP, torsade de pointes arrhythmia; EB, ectopic beat; NA, not applicable; TdP score, the number of beats in the TdP or 50/75/100 points when requiring 1, 2 or ≥3 defibrillations respectively of the most severe episode if > 1 TdP occurred.

**FIGURE 3:**
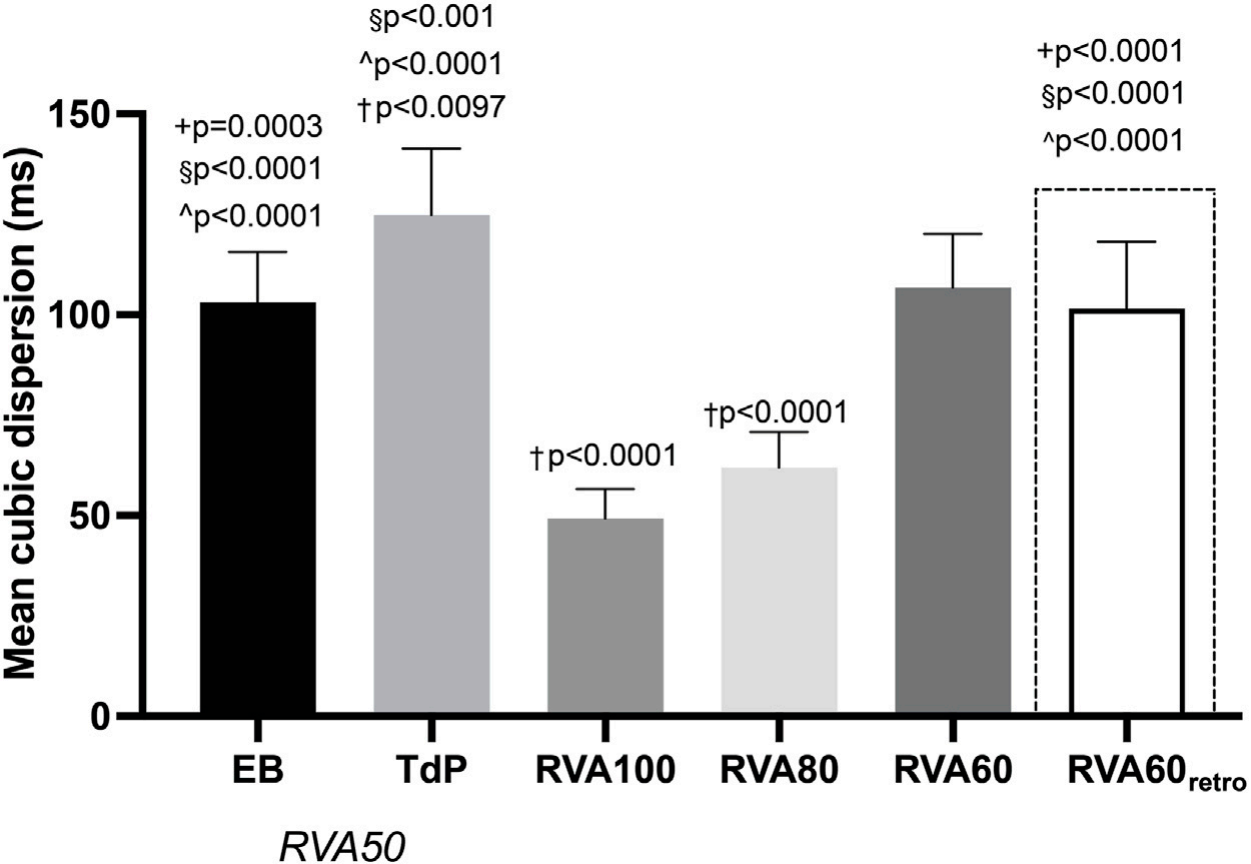
Progression of cubic dispersion of repolarization during different RVA pacing rates after dofetilide infusion in chronic atrioventricular block dogs. Values represented as mean ± SD. EB, ectopic beat; TdP, Torsade de Pointes arrhythmia; RVA, right ventricular apical pacing. *p < 0.05 vs. EB, ^+^ *p* < 0.05 vs. TdP, ^§^ *p* < 0.05 vs. RVA100, ^*p* < 0.05 vs. RVA80, †*p* < 0.05 vs. RVA60.

**FIGURE 4:**
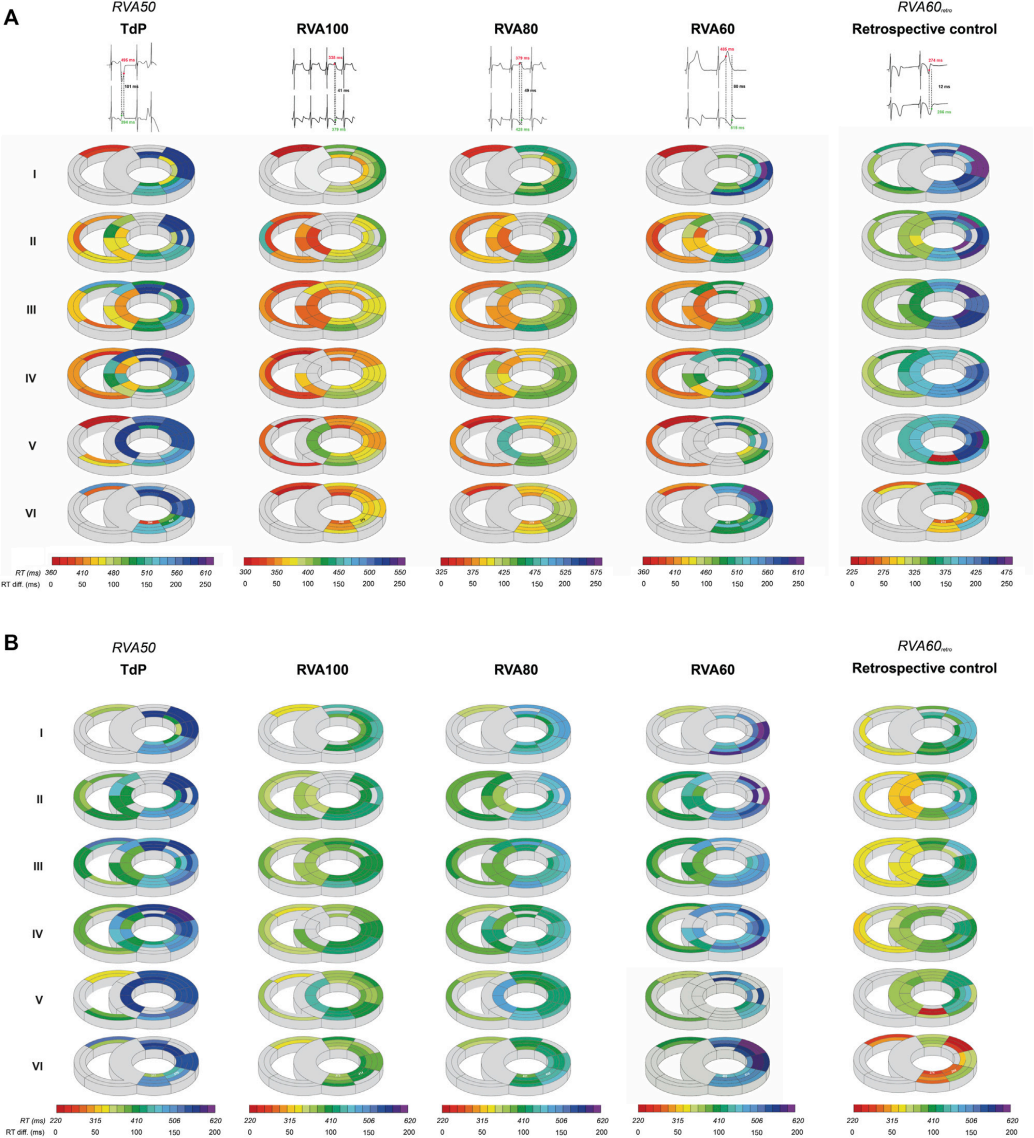
High-rate pacing decreases *intra*ventricular spatial dispersion in repolarization after dofetilide infusion. **(A)** A representative example of local RT in the RV, LV and septum from base (I) to apex (IV) derived from one animal during RVA50, RVA100, RVA80 and RVA60 pacing, in addition to retrospective control data at RVA60 pacing shortly after defibrillation. The evolution of spatial dispersion is shown using two unipolar electrogram traces along with their corresponding RT and their differences. Colors and gradients in the illustrations represent absolute RT and ΔRT values, respectively, as indicated in the color bars below the polar maps. **(B)** RTs of **(A)** corrected by the van de Water formula (RTc) illustrate that, although less pronounced, the difference in spatial dispersion can still be observed over varying pacing conditions. RT, repolarization time; RTc, corrected repolarization time; RV, right ventricle; LV, left ventricle; TdP, Torsade de Pointes; RVA, pacing from the right ventricular apex.

QT and QTc were similar prior to the first EB and TdP, but RV-RT and LV-RT prolonged significantly before TdP (Table 1). Moreover, this progression from EB to TdP was paralleled by an increase in SDR (Table 1). Increasing the pacing rate after TdP conversion shortened repolarization as demonstrated by QTc, RV-RT and LV-RT and significantly decreased SDR (Table 1). This effect was most prominent in the cubic orientation: SDR_cubic_ increased significantly from EB to TdP, decreased significantly during RVA100 and RVA80 pacing, and at RVA60 returned to values alike before the first EB (Figure 3). Figure 4 depicts local RT and local RT differences in the RV, LV and septum from base (I) to apex (IV) in the heart during different pacing regimens. Figure 4A shows the decrease in SDR during HRP. After correction of RT by the van de Water formula, the modulatory effect of HRP on SDR, although less pronounced, can still be observed (Figure 4B).

**FIGURE 5:**
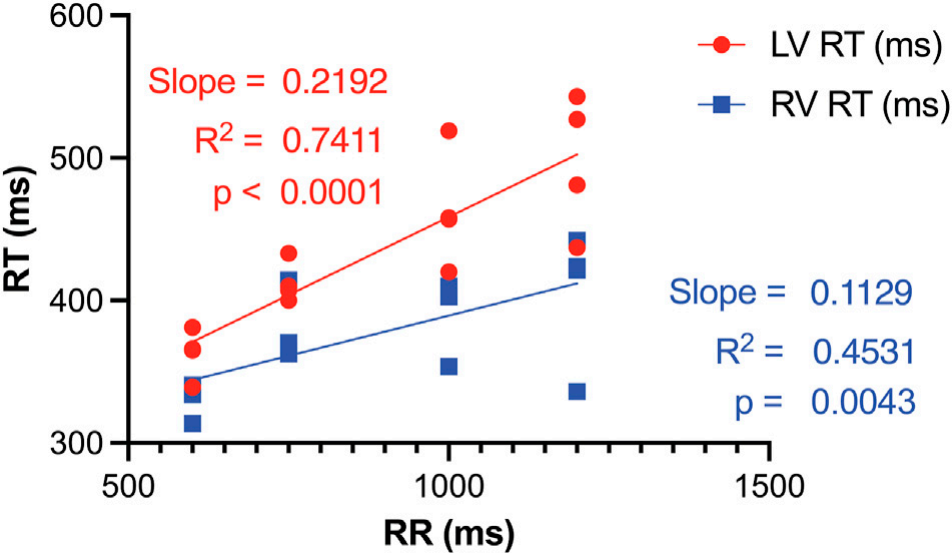
RT restitution curves of LV and RV illustrate that a reduction in pacing rate, i.e., an increase in RR, results in *inter*ventricular dispersion of RT. This occurs as the repolarization duration of the LV prolongs more significantly compared to the RV. RT, Activation recovery interval; LV, left ventricle; RV, right ventricle.

RT restitution curves displayed notable disparities between the LV and RV. The steeper slope in the LV curve elucidates a greater extent of RT prolongation in response to lower pacing rates (Figure 5). Consequently, *inter*ventricular dispersion in repolarization is smaller at higher pacing rates (Figure 5; Table 1). As such HRP contributes to the reduction of *inter*ventricular dispersion of repolarization, in addition to its role in diminishing repolarization duration and SDR.

Retrospective control animals were similarly inducible as the animals in the current study (at RVA60, time to first EB/TdP: 117 ± 10/230 ± 37 s after dofetilide infusion, respectively). However, 66% of these animals had reoccurrence of TdP, whereas none of the RVA100 animals displayed an episode of TdP. Moreover, both RV and LV-RT were significantly longer than in RVA100 (Table 1) and SDR was significantly higher (SDR_cubic_ 101 ± 52 in RVA60_retro_ vs*.* 49 ± 18 ms in RVA100, *p* < 0.0001). Interestingly, comparing RVA60_retro_ with RVA60 of the current data showed that RV-RT and LV-RT were significantly shorter in the historical data. However, SDR was not significantly different between the two groups, nor were arrhythmic outcomes.

## Discussion

The present study established the connection between HRP, SDR, and the occurrence of TdP in the CAVB dog model. It reaffirmed that accelerated pacing curbs arrhythmia development and mitigates event severity. This study is the first to show *in vivo* that this suppressive effect is, at least in part, a result of decreased SDR. Retrospective control data validate that decreased SDR and improved arrhythmic outcomes with RVA100 pacing stem from the influence of rapid pacing and not from recent defibrillation. Hence, this research highlights the anti-arrhythmic properties of HRP through modulation of the arrhythmic substrate.

### Role of SDR in arrhythmogenesis

The CAVB-model is an established model for the study of human TdP. Leading up to TdP, temporal variability in repolarization, quantified as STV, increased prior to the first EB, but remained stable afterwards (Smoczynska et al., 2021). In contrast, SDR elevated significantly in the development from EB to TdP (Smoczynska et al., 2021). This suggests that temporal dispersion of repolarization contributes to the initiation of arrhythmic events, while SDR gains prominence in perpetuating these events (van Weperen et al., 2019; Smoczynska et al., 2021). Further supporting this notion, it has been discovered that brief TdP episodes originate from and are sustained by localized activity, whereas persistent TdP rely on re-entry mechanisms (Vandersickel et al., 2017; Rivaud et al., 2021). These insights emphasize the theory that spatial repolarization heterogeneity serves as a functional substrate for perpetuation of ventricular arrhythmias. Several other studies underscore the significance of SDR in TdP development. Following dofetilide administration, dogs prone to TdP displayed a more notable increase in SDR compared to non-susceptible dogs (Dunnink et al., 2017). Additionally, this study showed that the beat triggering TdP could be traced back to the site with the highest SDR (Dunnink et al., 2017). Correspondingly, reducing SDR by the antiarrhythmic compound GS-458967 (I_Na-l_ inhibitor) completely suppressed TdP, even though single and multiple ectopic beats persisted and other electrophysiological parameters, including RT and STV, remained elevated (Bossu et al., 2018). Van Weperen *et al.* expanded these findings by illustrating that the antiarrhythmic efficacy of four potent anti-arrhythmic drugs was most accurately mirrored by SDR (van Weperen et al., 2019), while parameters based on repolarization duration only lacked sensitivity. The present study aligns with the notion that SDR outperforms RT as indicator of arrhythmic risk. RVA100 pacing abolished TdPs, which was accompanied by attenuation of the dofetilide-induced increase in SDR. Lowering pacing rates (VVI80 and VVI60) resulted in recurrence of TdP, higher ectopic burden and higher SDR (Table 1; Figures 2–4). The absence of a significant difference in arrhythmic outcomes between the RVA60 and RVA60_retro_ data is reflected by comparable SDR values. RT on the other hand did differ between RVA60 and RVA60_retro_ data, underlining the added value of SDR over RT for arrhythmic risk assessment. It is worth noting however that there could be an interaction between SDR and RT, as from a physiological point of view it makes sense that dispersion decreases as action potential duration shortens and since RT decreased upon faster pacing rates (Table 1). Figure 4B on the other hand demonstrates that the homogenizing effect of HRP is maintained after correction of RT for heart rate.

### Antiarrhythmic properties of elevated heart rate

This study demonstrated that HRP suppressed TdP by decreasing both *inter* (Figure 5) and *intra*ventricular dispersion of repolarization (Figures 3, 4; Table 1). The anti-arrhythmic effects of HRP had already been well-established in both clinical and experimental settings. Guidelines recommend accelerated pacing therapy to prevent ventricular arrhythmias in patients with long QT-syndrome and pause-dependent ventricular arrhythmias (Eldar et al., 1987; Fisher et al., 1987; Gronefeld et al., 2002; Priori et al., 2015). This principle is supported by several studies in the CAVB dog model, where high-rate pacing prevented the development of TdP arrhythmias in 70% of the dogs. These animals were paced at a rate of 100–110 bpm from the start of the arrhythmia induction protocol and upon the occurrence of the first ectopic beat respectively (Oosterhoff et al., 2010; Wijers et al., 2017). Correspondingly, severe bradycardia was shown to increase the probability of arrhythmia occurrence and episode severity in CAVB dogs, accompanied by an elevated SDR (van Weperen et al., 2021). Prior studies reported an increased *inter*ventricular dispersion of repolarization under bradycardic conditions and demonstrated its significance in the development of TdP as well (Restivo et al., 2004; Kim et al., 2013). In addition, HRP does not only decrease SDR, but also STV. A preclinical study showed that when HRP was automatically initiated by an implantable cardiac device upon surpassing a pre-programmed STV-threshold (Smoczynska et al., 2020a), STV decreased. This suggests that rapid pacing, in addition to attenuating a functional arrhythmic substrate, suppresses temporal dispersion in repolarization that is associated with triggering of ventricular arrhythmias.

Nevertheless, the mechanisms underlying the anti-arrhythmic properties of an elevated heart rate are complex and incompletely understood, though several additional mechanisms have been put forward. First, TdP are often preceded by a long–short sequence and it has been hypothesized that preventing these sudden rate changes through pacing would subsequently prevent initiation of ventricular arrhythmias (Viskin et al., 2000). Moreover, a shorter diastolic interval provides less time for the deactivation of I_Ks_, resulting in shortening of the action potential duration (Rocchetti et al., 2001). The repolarization reserve is further strengthened through amplification of the I_Kr_ current and inactivation of the L-type calcium current (Damiano and Rosen, 1984; Szentandrassy et al., 2015). These adaptations inhibit the formation of early and delayed after depolarizations, which can both trigger as well as perpetuate arrhythmias trough repolarization heterogeneities.

### Tachypacing vs*.* defibrillation

Defibrillation often leads to re-initiation of the arrhythmia (Chen et al., 1986). We show that RVA100 pacing leads to a stable suppression of TdP and low SDR 2 min following defibrillation, while RVA60 demonstrated recurrence of TdP and high SDR (Table 1; Figures 2–4). Moreover, the similarity in SDR between RVA60_retro_ and RVA60 after HRP demonstrates the limited effects of defibrillation on SDR.

### Clinical implications

Comprehending the direct impact of heart rate on cardiac electrophysiology is crucial, encompassing its dual role in fostering and preventing the formation of ventricular arrhythmias during bradycardic and tachycardic circumstances, respectively. Particularly the latter observation holds clinical significance, as it underscores the potential utility of pacemakers in managing cardiac diseases with an elevated risk of ventricular arrhythmias. Since the current work demonstrated that rapid pacing modulates a functional arrhythmic substrate, in addition to triggering events as demonstrated before (Wijers et al., 2017; Smoczynska et al., 2021), it suggests that this approach could be useful for a broad range of cardiac conditions. Furthermore, this study demonstrated complete suppression of arrhythmias at a pacing rate of 100 beats per minute, whereas pacing at 80 beats per minute only partially subdued ventricular arrhythmias. This highlights how a faster pace, specifically 100 beats per minute, emerges as the most efficacious approach for preventing ventricular arrhythmias in the CAVB dog model. However, since chronic right-ventricular pacing has been demonstrated to jeopardize LV function, resulting from ventricular remodeling induced by asynchronous activation, HRP should be utilized only in cases where a distinct arrhythmic threat is evident (Tops et al., 2009; van Weperen et al., 2023).

While the dog has been recognized as the species with the highest predictability in cardiac electrophysiological studies, it is important to acknowledge the physiological differences between dogs and humans (Clauss et al., 2019; Loen et al., 2022). These differences encompass factors like varying impacts of repolarizing currents and different function of the autonomic nervous system (Jost et al., 2013; Fukuda et al., 2015).

### Limitations

Altered ventricular activation through RVA pacing is known to influence electrical stability and promote pro-arrhythmic remodeling (van Weperen et al., 2023). Nevertheless, the anti-arrhythmic effect of elevated pacing was clearly demonstrated and pacing within each dog was carried out from an identical endocardial site, ensuring the reliability of serial comparisons.

Furthermore, dofetilide infusion was stopped prematurely when TdP occurred within 5 min after infusion. Therefore, differences in dofetilide administration exist between the dogs. On the other hand, RV-RT and LV-RT were significantly longer during RVA60 of the current data than in the RVA60_retro_ data, posing a counterargument for possible subtherapeutic dofetilide levels at the end of the protocol of the current study. Despite the expectation of a reduced occurrence of TdPs based on the lower RTs in the RVA60_retro_ data, there were no statistically significant differences found in arrhythmic outcomes and SDR, underlining the added value of SDR in risk assessment.

Finally, the spacing between electrodes was smaller in a single needle (4 mm) compared to the spacing of electrodes between needles (1 cm). As a result, the measurements of SDR taken in various orientations cover varying distances.

## Conclusion

High-rate pacing effectively suppresses the development of polymorphic ventricular arrhythmias in the CAVB dog, which is, at least in part, a result of homogenization of cardiac repolarization duration. Our data suggest that HRP is a potential adjuvant to defibrillation alone.

## Data Availability

The raw data supporting the conclusion of this article will be made available by the authors, without undue reservation.
